# Understanding Patients’ Preferences for a Digital Intervention to Prevent Posttreatment Deterioration for Bulimia-Spectrum Eating Disorders: User-Centered Design Study

**DOI:** 10.2196/60865

**Published:** 2024-11-18

**Authors:** Jianyi Liu, Alyssa Giannone, Hailing Wang, Lucy Wetherall, Adrienne Juarascio

**Affiliations:** 1 Department of Psychological and Brain Sciences Drexel University Philadelphia, PA United States; 2 Center for Weight, Eating and Lifestyle Sciences Drexel University Philadelphia, PA United States; 3 Graduate School of Education University of Pennsylvania Philadelphia, PA United States

**Keywords:** bulimia nervosa, binge eating, digital intervention, deterioration prevention, eating disorder, bulimia, digital health, deterioration, maintenance, mHealth, mobile health app, interviews, qualitative, user-centered design, psychotherapy, CBT, cognitive behavioral therapy, needs, preferences, mobile phone

## Abstract

**Background:**

Deterioration rates after enhanced cognitive behavioral therapy (CBT-E) for patients with bulimia-spectrum eating disorders (BN-EDs) remain high, and decreased posttreatment skill use might be a particularly relevant contributor. Digital interventions could be an ideal option to improve skill use after treatment ends but they have yet to be investigated for BN-EDs.

**Objective:**

This study used a user-centered design approach to explore patients’ interest in a digital intervention to prevent deterioration after CBT-E and their desired features.

**Methods:**

A total of 12 participants who previously received CBT-E for BN-EDs and experienced at least a partial response to treatment completed a qualitative interview asking about their interests and needs for an app designed to prevent deterioration after treatment ended. Participants were also presented with features commonly used in digital interventions for EDs and were asked to provide feedback.

**Results:**

All 12 participants expressed interest in using an app to prevent deterioration after treatment ended. In total, 11 participants thought the proposed feature of setting a goal focusing on skill use weekly would help improve self-accountability for skill use, and 6 participants supported the idea of setting goals related to specific triggers because they would know what skills to use in high-risk situations. A total of 10 participants supported the self-monitoring ED behaviors feature because it could increase their awareness levels. Participants also reported wanting to track mood (n=6) and food intake (n=5) besides the proposed tracking feature. A total of 10 participants reported wanting knowledge-based content in the app, including instructions on skill practice (n=6), general mental health strategies outside of EDs (n=4), guided mindfulness exercises (n=3), and nutrition recommendations (n=3). Eight participants reported a desire for the app to send targeted push notifications, including reminders of skill use (n=7) and inspirational quotes for encouragement (n=3). Finally, 8 participants reported wanting a human connection in the app, 6 participants wishing to interact with other users to support and learn from each other, and 4 participants wanting to connect with professionals as needed. Overall, participants thought that having an app targeting skill use could provide continued support and improve self-accountability, thus lowering the risk of decreased skill use after treatment ended.

**Conclusions:**

Insights from participants highlighted the perceived importance of continued support for continued skill use after treatment ended. This study also provided valuable design implications regarding potential features focusing on facilitating posttreatment skill use to include in digital deterioration prevention programs. Future research should examine the optimal approaches to deliver the core features identified in this study that could lead to higher continued skill use and a lower risk of deterioration in the long term.

## Introduction

Bulimia-spectrum eating disorders (BN-EDs), characterized by recurrent binge eating episodes (ie, eating a large amount of food within a discrete period of time with a sense of loss of control [LOC]) and compensatory behaviors (eg, self-induced vomiting, compensatory laxatives use, and driven exercise), are associated with significant medical consequences and psychological comorbidities [1]. Enhanced cognitive behavioral therapy (CBT-E) is currently the gold-standard treatment for patients with BN-EDs, with 30% of patients achieving remission (ie, cessation of binge eating and compensatory behaviors) at the end of treatment (EOT) and up to 70% achieving a large reduction in symptoms [2,3]. However, over 30% of patients who achieve remission would relapse (ie, reoccurrence of symptoms after remission) within 12 months of completing CBT-E [4], and the deterioration (ie, worsening of symptoms after responding to treatment by experiencing a large reduction in symptoms, defined in the Methods section) rate is likely higher for patients who are not fully remitted at EOT [4,5]. Relapse and deterioration are strongly associated with worsened quality of life [6,7] and could lead to patients being readmitted to treatment, adding burden and cost to both patients and health care systems [8].

Despite high rates of deterioration, there are currently no deterioration prevention programs available for patients with BN-EDs receiving outpatient CBT-E to address this concern. One factor that may be particularly relevant to posttreatment deterioration is patients’ posttreatment skill use. CBT-E is a highly skill-focused treatment that teaches patients 6 core skills, including regular eating (eating every 3-4 waking hours), eating enough food and macronutrients (at least 3 macronutrients for meals and 2 for snacks), incorporating feared foods and binge trigger foods back into daily diets, learning to identify triggers to binge eating, using coping strategies to manage negative emotions, and managing urges to engage in ED behaviors (eg, binge eating, self-inducing vomiting, and skipping meals) [2]. More frequent skill use during CBT-E has been associated with better cognitive (eg, excessive concerns about body weight and shape and body dissatisfaction) and behavioral treatment outcomes (eg, binge eating episodes) among patients with BN-EDs [9-11]. However, after finishing treatment, patients might experience a decrease in skill use due to a lack of self-accountability, meaning that they might not be able to practice the skills on their own without outside reminders or encouragement, which has been shown to be a core contributor to the deterioration [12]. Therefore, having a skill-use-focused deterioration prevention could potentially be helpful for patients with BN-EDs after receiving outpatient CBT-E.

Additionally, limited access to resources including mental health services has been identified as another factor associated with posttreatment deterioration for patients with BN-EDs [12,13]. Patients have indicated a need for continued guidance and support to remind and encourage them to keep practicing the skills, otherwise they would lose self-accountability to do so, especially when they encountered challenging situations [12-14]. For example, patients have noted that losing the regular check-ins with their therapists made them stop practicing mood and urge management skills after treatment ended, as they felt overwhelmed by the negative emotions and urges and chose to turn back to binge eating as it was an easier solution [12]. Low-burden digital deterioration prevention might be an ideal option to address this concern. Digital interventions, compared to in-person interventions, could facilitate skill use in patients’ home environments, personalize intervention contents and delivery systems, and are more cost-effective [13]. Much of the past literature on digital relapse and deterioration prevention programs for EDs targeted patients receiving treatment at higher levels of care [15-18]. Additionally, these digital interventions were also similar in intensity to outpatient ED treatment, typically consisting of high-intensity self-monitoring, long psychoeducational modules, and regular therapists’ involvement. Further, existing digital relapse and deterioration prevention programs for EDs only yielded extremely small effect sizes [19,20], which might be associated with higher baseline eating pathology (participants discharged from inpatient treatment), long program duration (3-9 months), and low compliance rates [20]. Thus, effective and low-burden digital deterioration prevention is urgently needed for the outpatient BN-EDs population.

User-centered design (UCD, also referred to as human-centered design) is an important approach to use when conceptualizing and designing a digital intervention [21]. UCD is an iterative process that always puts the core users at the center when designing a digital product. The first step is to learn core users’ needs and preferences (Investigate), generate initial product ideas (Ideate), create a minimally viable product as a prototype (Prototype), iteratively test the prototype (Evaluate), refine the design and develop the final product (Refine and Develop), and test the product in real-world (Validate) [22]. Applying a UCD approach helps maximize the usability and effectiveness of a digital intervention by deliberately considering patients’ needs and goals throughout the design process [22,23]. Therefore, to first conceptualize digital deterioration prevention for patients with BN-EDs after receiving CBT-E, it is essential to conduct qualitative needs assessments with them to gather detailed information regarding their needs and preferences so that the final product will fit well with patients’ goals, which could lead to higher patient satisfaction, higher patient engagement, and eventually better treatment outcomes [22,24].

Thus, to improve the interventions offered to patients with BN-EDs and better prevent deterioration after receiving CBT-E, there is a need to first understand patient perspectives regarding digital app interventions targeting deterioration prevention. For this study, qualitative interviews were conducted with 12 patients with BN-EDs who had previously received 16 sessions of CBT-E and experienced symptom improvement at EOT. These qualitative interviews aimed to use a UCD approach, specifically in the Investigate and Ideate phases, and explore: (1) patients’ interests and needs for a digital app intervention designed to prevent deterioration after finishing CBT-E and (2) patients’ preferences on features they thought would be beneficial to be included in the app for improving skill use and preventing deterioration.

## Methods

### Participants and Procedures

A total of 56 participants who were diagnosed with BN-EDs received outpatient CBT-E in a prior clinical trial at Drexel University [25]. To be eligible for this study, participants needed to have completed all 16 sessions of CBT-E in the previous trial and responded to treatment by experiencing improvement from baseline to EOT in at least one of the following areas: (1) a reduction of at least 0.5 in eating disorder examination (EDE; see detailed description in Measures) global score, (2) a decrease of at least 50% in the frequency of LOC eating episodes, or (3) a decrease of at least 50% in the frequency of compensatory behaviors. The cognitive criteria (ie, global eating pathology including concerns about eating, weight, and shape; EDE global score decreased by 0.5) was used in the study by Kim et al [26]. Kim et al [26] defined meaningful treatment response as a decrease in EDE global scores by SD 0.5. Given that the original sample in the study by Juarascio et al [25] had an SD of 1.00 for the EDE global score, this study adopted 0.5 as the inclusion criterion. The behavioral criteria (ie, LOC eating episodes and compensatory behaviors decreased by 50%) paralleled definition of Carter et al [27] of meaningful treatment response, which was conceptualized as a 50% reduction in bulimic symptoms. Study staff from this study analyzed participants’ baseline and posttreatment data in the prior clinical trial, and based on the inclusion criteria, 42 participants from the prior clinical trial were deemed eligible for this study.

All 42 eligible participants received a recruitment email, which stated the purpose of this follow-up study and explained the study procedures and compensation. Participants were also told that their participation would be completely voluntary. Of those, 12 participants expressed interest and participated in the study (mean age 40.08 years, SD 13.52 years). Table 1 presents detailed characteristics of these participants. All participants had previously received a diagnosis of BN in the prior clinical trial. By EOT, 4 participants achieved complete remission, and the remaining 8 participants achieved meaningful treatment responses. The duration between the participants’ completing treatment and this study varied between 34.22 and 49.95 months, with an average follow-up duration of 39.85 (SD 3.95) months. Participants participated in the study via two Zoom (Zoom Video Communications, Inc) sessions. In the initial session, participants provided consent, answered demographic questions, and completed the EDE. Subsequently, they were asked to fill out a self-report survey asking about their posttreatment experiences and utilization of CBT-E skills. The second session involved participants engaging in a qualitative interview facilitated by a clinical psychology PhD student (JL). Table 1 presents the average EDE global score and the number of LOC eating episodes and compensatory behaviors during both the parent study and the time of this study. Nine participants exhibited deterioration, defined as experiencing an increase in EDE global score by 0.5, a 50% increase in past-month LOC eating episodes, or a 50% increase in past-month compensatory behaviors compared to EOT. Among the remaining three participants, two participants reported during the qualitative interview a temporary deterioration posttreatment, followed by subsequent improvement. Therefore, 11 out of the 12 participants experienced deterioration at some time since EOT.

**Table 1:** Participants’ characteristics, including demographics by the time of this study, global eating pathology, and number of past-month eating disorder behaviors at baseline, post treatment, 3-month follow-up, and this study.

Characteristics		Value (N=12)
**Age (years)**		
	Mean (SD)	40.08 (12.52)
	Range	25-65
BMI, mean (SD)		29.74 (7.99)
**Race, n (%)**		
	Asian	1 (8)
	Black	1 (8)
	Mixed Race	2 (17)
	White	7 (58)
	Other	1 (8)
**Ethnicity, n (%)**		
	Hispanic	1 (8)
	Non-Hispanic	11 (92)
**EDE^a^ global scores, mean (SD)**		
	Baseline	3.11 (0.83)
	Posttreatment	1.19 (0.72)
	3-month follow-up	1.13 (1.01)
	This study	1.94 (1.01)
**Past-month LOC^b^ eating episodes, mean (SD)**		
	Baseline	16.08 (9.68)
	Post treatment	2.25 (3.89)
	3-month follow-up	4.42 (9.61)
	This study	4.83 (5.83)
**Past-month compensatory behaviors, mean (SD)**		
	Baseline	24.58 (18.02)
	Post treatment	3.92 (10.82)
	3-month follow-up	5.17 (10.36)
	This study	7.5 (13.57)

^a^EDE: eating disorder examination.

^b^LOC: loss of control.

### Measures

#### Demographics

In the demographic questionnaire, participants reported their age, race, ethnicity, weight, height, current medications, and ongoing mental health treatment.

#### EDE

The EDE [28] is a semistructured clinical interview that was used in this study to assess participants’ eating pathology and ED behaviors over the past 28 days. It generates 4 subscales, including Restriction, Eating Concerns, Weight Concerns, Shape Concerns, and a global score (ie, the average of the four subscales). This study used the global score to assess participants’ global eating pathology. Global scores can range from 0 to 6, with higher scores indicating more severe eating pathology. This study also used the EDE to assess the number of binge-eating episodes and compensatory behaviors (ie, self-induced vomiting, laxative use, diuretic use, driven exercise, and other extreme weight control behaviors) within the past 28 days.

#### Qualitative Interview

##### Overview

The qualitative interview was designed to ask about participants’ interests in and needs for digital deterioration prevention right after treatment ended. All participants were asked to think back to the time when they were about to finish treatment, whether they would be interested in using an app designed specifically for this purpose, and what features they would like to have. Then, three specific features, weekly ED behaviors self-monitoring, weekly skill-focused goal-setting with personalized feedback, and weekly skill-focused goal-setting for specific triggers, were proposed to all participants for their feedback. These features were chosen because they are grounded in the CBT framework and have been widely used in digital interventions for EDs [29,30]. Participants were also presented with graphic descriptions of each feature during the qualitative interview (all of which are available in Multimedia Appendix 1). Table 2 presents the specific questions used in the qualitative interview.

**Table 2:** Qualitative interview questions for all participants.

Section	Interview questions
Needs assessment for digital intervention to prevent deterioration	For this section, we will be asking you about your interests and needs for a digital app intervention designed to prevent ED^a^ symptoms deterioration after treatment has ended. First, if you think back to the time when you were about to finish treatment, would you have been interested in using an app after treatment ended? (If yes) How do you think digital health tools, like smartphone apps, could have been helpful for you? What specific features would you like to have in the app? Why? What are some barriers you anticipate might interfere with your engagement with the app? How often do you anticipate you might use such tools? (If no) Why not?
Feedback on proposed digital intervention features	For the rest of this interview, we would like to gather your feedback on some potential features. One of the features is weekly symptom monitoring. This feature will require you to report your eating disorder symptoms weekly, including the number of loss of control episodes and compensatory behaviors. Based on your response, you will be presented with a graph of your symptom trajectory. What is your overall impression of this feature? How burdensome is weekly self-monitoring? How do you like the frequency of weekly self-monitoring? Is it not enough, too much, or just enough? Do you think this feature would be helpful? How? What are some potential barriers that you anticipate might get in the way of using this feature on a regular basis? Another feature is weekly goal setting and personalized feedback. The goal-setting feature will require you to set 3 goals at the beginning of each week, for example, for this week, I will keep eating regularly with 3 meals and 2 snacks every day. After setting the goals, you will choose the strategies you want to practice for the week, for example, planning for snacks ahead of time. At the end of the week, you will report how well you achieved the goal for the week, and whether the strategies were useful. In addition, at the beginning of the week, a message with personalized feedback will be sent to you based on your last week’s performance on your goals. It will include a short summary of your performance, encouraging messages, and a reminder for you to set new goals for the week. It will also provide personalized recommendations on choosing strategies to practice. Sample feedback would be, “Last week, you have successfully achieved your goal (eat regularly with 3 meals and 2 snacks) on 5 days! Well done! Your strategy of planning snacks ahead of time has proven successful 80% of the time, and when you practiced it you have always found it helpful, great job! I encourage you to keep practicing this strategy for the upcoming week, as it has been essential in your progress. In addition, don’t forget to take some time to set new goals for the upcoming week, reflecting on your recent achievements and identifying areas for further growth.” What is your overall impression of this feature? How burdensome is weekly goal setting and checking-in? Do you think this feature would be helpful? How? What are some potential barriers that you anticipate might get in the way of using this feature on a regular basis? To target specific triggers, one feature, we will call it goal setting—trigger version here, is to incorporate that into the weekly goal setting. You will be asked to plan ahead of time what strategies you plan to use when encountering the triggers and set a goal based on your plan. For example, you can say stressful work events often trigger you to have the urge to binge, and you plan to use alternative activity and problem-solving strategies to deal with this trigger, and thus for this week, you will set a goal of “actively dealing with environmental triggers at work,” and the strategies to practice would be engaging in alternative activities and practicing proactive problem-solving. At the end of the week, you will also report how well you achieved the goal for the week, and whether the strategies were useful. What is your overall impression of this feature? How burdensome do you think this is? Do you think this feature would be helpful? How? What are some potential barriers that you anticipate might get in the way of using this feature on a regular basis?

^a^ED: eating disorder.

Prompts describing the features provided to participants are listed below.

##### Weekly ED Behaviors Self-Monitoring

**Figure 1:**
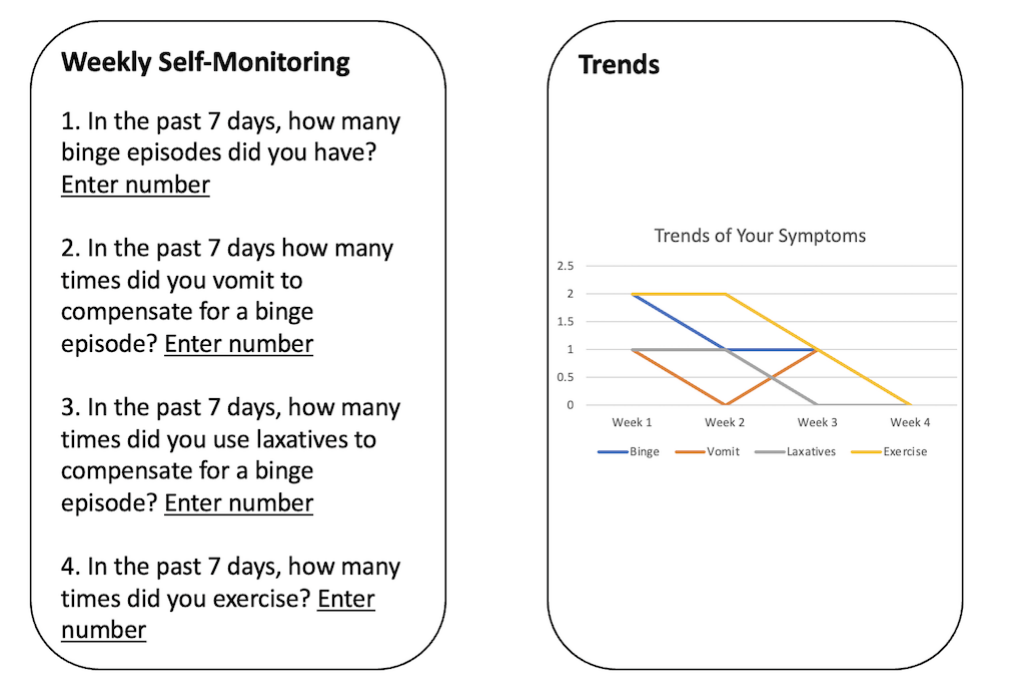
Graphic descriptions of weekly ED behaviors self-monitoring shown to participants during the qualitative interview to gather their feedback on this feature. ED: eating disorder.

One of the features is weekly symptom monitoring. This feature will require you to report your eating disorder symptoms weekly, including the number of LOC episodes and compensatory behaviors. Based on your response, you will be presented with a graph of your symptom trajectory (Figure 1).

##### Weekly Skill-Focused Goal-Setting With Personalized Feedback

**Figure 2:**
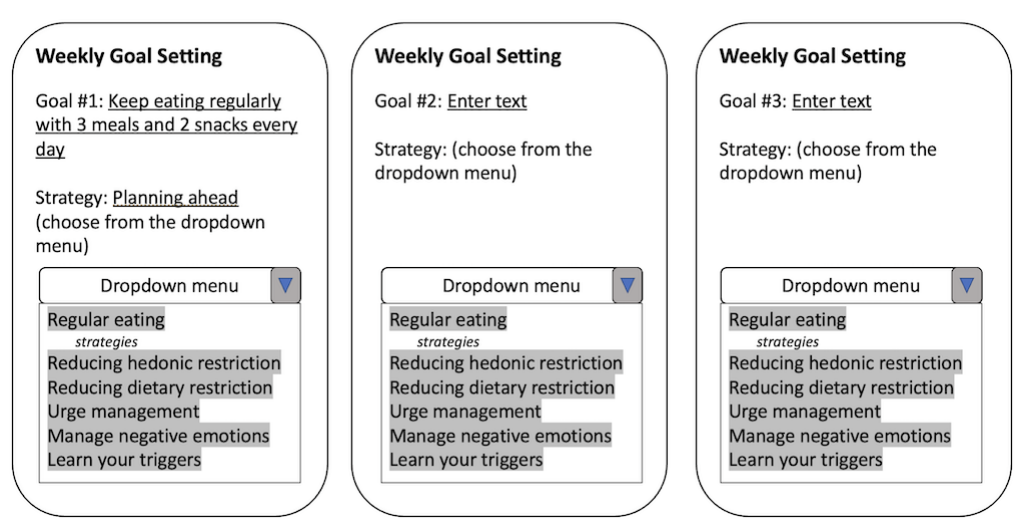
A graphic description of weekly skill-focused goal-setting shown to participants during the qualitative interview to gather their feedback on this feature.

Another feature is weekly goal setting and personalized feedback (Figure 2). The goal-setting feature will require you to set 3 goals at the beginning of each week, for example, “For this week, I will keep eating regularly with 3 meals and 2 snacks every day.” After setting the goals, you will choose the strategies you want to practice for the week, for example, planning for snacks ahead of time. At the end of the week, you will report how well you achieved the goal for the week, and whether the strategies were useful. In addition, at the beginning of the week, a message with personalized feedback will be sent to you based on your last week’s performance on your goals. It will include a short summary of your performance, encouraging messages, and a reminder for you to set new goals for the week. It will also provide personalized recommendations on choosing strategies to practice. Sample feedback would be, “Last week, you have successfully achieved your goal (eat regularly with 3 meals and 2 snacks) on 5 days! Well done! Your strategy of planning snacks ahead of time has proven successful 80% of the time, and when you practiced it you have always found it helpful, great job! I encourage you to keep practicing this strategy for the upcoming week, as it has been essential in your progress. In addition, don’t forget to take some time to set new goals for the upcoming week, reflecting on your recent achievements and identifying areas for further growth.”

##### Weekly Skill-Focused Goal-Setting for Specific Triggers

To target specific triggers, one feature, we will call goal setting—trigger version here, is to incorporate that into the weekly goal setting. You will be asked to plan ahead of time what strategies you plan to use when encountering the triggers and set a goal based on your plan. For example, you can say stressful work events often trigger you to have the urge to binge, and you plan to use alternative activity and problem-solving strategies to deal with this trigger, and thus for this week, you will set a goal of “actively dealing with environmental triggers at work,” and the strategies to practice would be engaging in alternative activities and practicing proactive problem-solving. At the end of the week, you will also report how well you achieved the goal for the week, and whether the strategies were useful.

### Analyses

An inductive approach was used for the qualitative data analysis. Two undergraduate research assistants transcribed and deidentified all interview recordings (Eli Wiener and Jaira Marcos). Four graduate students (JL, AG, HW, and LW) in psychology and related fields reviewed the transcripts and generated a preliminary set of codes (ie, detailed descriptions of features participants reported wanting and reasonings). Then, the preliminary codes were grouped into upper-level themes based on common functionalities (ie, categories of features). Coders coded proposed features and participant-generated features separately. Four transcripts were double-coded to ensure interrater reliability [31].

### Ethical Considerations

This study was approved by Drexel University’s institutional review board (protocol 2304009864). All participants were provided with an institutional review board–approved informed consent form before their first Zoom session. During the first Zoom session, the interviewer went through the informed consent form with the participants and answered all of the questions participants had, and all participants electronically signed the informed consent form before completing any study procedures. All participants’ data were deidentified and stored on secured servers in password-protected files and folders. Participants received US $75 through QuickPay, an electronic payment system, to compensate for their time.

## Results

### Overview

All 12 participants expressed interest in using an app to prevent deterioration after treatment ended. They believed the app could provide continuous support and accountability for skill use (n=12) and help them monitor progress and learn from patterns (n=11). Table 3 presents individual features participants reported wanting to have in the app during the qualitative interview. Based on reviewing participants’ responses, 5 key features emerged based on the criteria of equal to or more than half of the participants endorsing the feature.

**Table 3:** Main categories of features and specific features mentioned by participants during the qualitative interview and representative quotes.

Features			Quotes
**Skill-focused goal setting with personalized feedback (n=11)**			
	Skill-focused goal setting for specific triggers (n=6)	“Because having a goal helps you succeed, and then, having a strategy gives you the action that you need to take to meet the goal.” “I think three goals are too much. Life is busy itself with work, relationships, kids, family, to think in your head. Three things I think it’s too much. Maybe cut it down.”	
**Self-monitoring**			
	ED^a^ behaviors (n=10) Mood (n=6) Food intake (n=5) Bedtime (n=1)	“I’m a big data person, so the more data you have, you can go and analyze it and see what patterns and triggers were there. So, I think it would help analyze those patterns and identify those triggers.” “I guess the only thing I would be interested in is real-time tracking of binge episodes, but the reflecting wouldn’t be helpful to me ... just feels like it’s very research-oriented versus about the person.”	
**Knowledge**			
	CBT-E^b^ skills (n=6) General mental health strategies outside of ED (n=4) Guided mindfulness or meditation exercises (n=3) Nutrition recommendations (n=3) Research evidence behind the skills (n=1)	“You can click in and get more detail, because I forgot what is reducing hedonic restriction. I totally forgot that. So you have to be able to click through and get access to something that spells it all out. So the details about how you would practice this.”	
**Notifications**			
	Reminders of skill use (n=7) Inspirational quotes for encouragement (n=3)	“I think it would be helpful if I’m in a situation, and I get some kind of alert saying, you know, why don’t you try these methods ... The barrier would be knowing when to send the notification.”	
**Human connection**			
	Connect with other users of the app (n=6) Connect with professionals (n=4) Any type of human connection (n=3)	“You would need some kind of community support especially for the failures. I know people get into a cycle of failure where you know, they just spiral down and so, having a community, or a therapist, or someone to review (the failures), I think would be great.”	

^a^ED: eating disorder.

^b^CBT-E: enhanced cognitive behavioral therapy.

### Goal-Setting With Personalized Feedback

A total of 11 participants supported the proposed weekly skill-focused goal-setting with a personalized feedback feature, which asked them to set 3 goals related to different CBT-E skills weekly. However, 6 participants thought that setting three goals a week was too burdensome and that they would prefer to set one goal per week. Participants who supported the weekly goal-setting feature stated that setting a goal to practice a certain skill regularly would help improve self-accountability for skill use. Six participants supported the feature of setting goals on how to handle specific triggers. These participants all mentioned that having this feature could be helpful for them to know what skills to use to target specific triggers related to high-risk situations (eg, work-related stress). Regarding the personalized feedback about goal achievement and effectiveness of skill use, 10 participants reported liking this feature. Among these participants, 6 participants thought that having feedback on goal achievement was encouraging and could serve as positive reinforcement, 5 participants stated that having specific metrics measured and presented to them could improve self-accountability to achieve the goals, and 3 participants reported the feedback would be a nice reminder to set new goals for the next week. Participants also identified several barriers to fully engaging with goal-setting, including lack of external support (n=3), forgetting details about specific strategies (n=1), and setting unachievable goals (n=1). To mitigate these barriers, participants also proposed several ideas including adding a community feature to provide accountability (n=1), tracking goal achievement every day (n=1), and having push notifications as reminders (n=1). Regardless of the perceived barriers, the proposed weekly goal-setting feature was overall perceived as helpful in facilitating skill use by improving self-accountability.

### Self-Monitoring

Ten participants reported wanting the proposed weekly self-monitoring ED behaviors feature, which asked them to self-report the number of LOC eating episodes and compensatory behaviors at the end of the week, because it could make them more aware of their ED behaviors and use relevant skills to address them. Regarding the frequency of self-monitoring, 7 participants thought that weekly monitoring would be just enough, 3 participants reported wanting to monitor daily, and 1 participant stated that the frequency should be customizable based on personal preferences. Two participants did not support the weekly self-monitoring feature, as it seemed research-oriented rather than patient-centered (n=2), and it was depressing to log ED behaviors (n=1). Participants who liked this feature also mentioned several barriers that might impact their engagement with self-monitoring, including lack of consistency without reminders and poor internet connection, and push notification reminders were proposed as a potential solution. In addition to the proposed self-monitoring feature, participants also had their ideas about what they wanted to monitor. Six participants reported wanting to monitor their mood daily, primarily to identify associations between mood and eating habits. Five participants reported wanting to monitor their food intake daily so that they could identify eating patterns. Overall, participants had various reactions to the proposed self-monitoring features, including wanting different monitoring frequencies and targets, but the majority of them supported the utility of self-monitoring to keep a high awareness level of their ED behaviors.

### Knowledge

Ten participants reported wanting knowledge-based content in the app. Six participants stated that having detailed instructions on how to use each skill would be helpful to refresh their memory and refamiliarize them with what they learned in treatment. Four of these participants reported that they would also like to have the skills organized based on their high-risk situations so that they could easily access the instructions when dealing with active triggers. Four participants expressed interest in learning more about strategies for their general mental health outside of ED, especially on coping with depression and anxiety. These participants thought that learning about general mental health strategies could be helpful both in dealing with comorbidities and in addressing ED symptoms tightly associated with them. In addition, three participants mentioned that they would like to have educational content on guided mindfulness exercises. Among them, 2 participants thought mindfulness exercises could help cope with negative emotions, and one participant thought they could help address negative thoughts related to body image. Finally, 3 participants reported wanting the app to have nutrition recommendations so that they could plan their meals and snacks more easily. In general, participants thought that having knowledge-based content could help enhance the use of skills learned in treatment, as well as provide opportunities to learn new skills, both of which were perceived as helpful in lowering the risk of deterioration of ED symptoms.

### Notifications

Eight participants reported a desire for the app to send targeted interventions via push notification. Specifically, 7 participants noted that having reminders of skill use would be helpful in encouraging consistent skill use, and five of them reported wanting the notification to be sent based on their skill use and ED behaviors. Three participants reported that they would like the app to send inspirational quotes for encouragement, and two of them thought having inspirational messages might be particularly helpful for coping with negative emotions triggered by body image concerns. Participants indicated an overall interest in having push notifications as reminders for different purposes in the app.

### Human Connection

Eight participants reported wanting a human connection in the app. Six participants reported wishing to interact with other patients with similar diagnoses who were also using the app so that the app could foster a social support system among them. These participants stated that connecting with other app users could make them feel less alone in the recovery process (n=3), celebrate and support each other (n=3), learn from other people’s success (n=2), and talk to someone who understood what they were going through during difficult times (n=2). Four participants reported wanting to connect with professionals for support and guidance when they experienced difficulty practicing skills. Specifically, three of these participants mentioned that negative emotions would be the primary challenge to skill use they would need professional help with. Overall, participants perceived human connection to be useful in both creating a supportive community with peers and providing access to proper help and guidance for skill use when in need.

## Discussion

### Principal Findings

This study qualitatively assessed patients with BN-EDs’ interests and needs for digital deterioration prevention after they finished CBT-E. All participants expressed interest in using such an app after treatment ended, stating that it could help with improving self-accountability for continued skill use, and monitoring and learning from progress. Participants also identified five key features they would like to include in the app that they believed could help with skill use and deterioration prevention.

The majority of the participants supported the proposed features of weekly goal-setting with personalized feedback and weekly ED behaviors self-monitoring. Self-monitoring and goal-setting are core features in face-to-face ED treatment [2] and many digital ED interventions delivered via smartphone apps [29]. Participants in this study noted that in the deterioration prevention phase, these features could potentially help increase awareness levels of ED behaviors and the need for consistent skill use, as well as increase self-accountability for independent skill use. Low awareness levels and low self-accountability have been identified as core factors contributing to deterioration in patients with BN-EDs [12]. Therefore, including these two features in an app designed specifically to prevent deterioration for patients with BN-EDs could align well with patients’ needs and potentially decrease the risk of deterioration. However, participants’ feedback on the frequency and targets of these features suggested that there was a need for flexibility and customization. For example, some participants stated an interest in monitoring food intake and mood daily, whereas other participants thought that monitoring ED behaviors weekly would be enough. Similarly, for goal-setting, some participants wanted to set more than one goal each week, while others thought that would be too burdensome. By allowing patients to adjust the frequencies and targets of self-monitoring and goal-setting to suit their personal preferences, the app could potentially have higher user satisfaction, higher user engagement, and thus better treatment outcomes [22].

Besides the proposed features, participants also reported several features they would like to have in the app. Many participants reported wanting to have knowledge-based content. A library of skills and strategies and detailed instruction on how to practice them was the most frequently mentioned among these participants, as it could help refresh their memory about the skills. Indeed, forgetting about skills and strategies learned in treatment is one of the contributors to decreased skill use and subsequent deterioration for patients with BN-ED after completing CBT-E [12]. Therefore, having knowledge-based content on skill use could also potentially help address this concern, thus lowering the risk of deterioration. In addition to skills learned in ED treatment, participants also reported wanting to have general mental health skills outside of EDs so that they could have tools to cope with comorbidities. Previous research has demonstrated that psychiatric comorbidities were associated with high risks of deterioration and relapse [32]. However, none of the existing digital deterioration prevention for ED has included such features. Therefore, digital deterioration prevention should incorporate both ED-specific knowledge-based content and knowledge about improving general mental health, as it could assist with preventing the deterioration of ED symptoms by potentially enhancing posttreatment skill use, as well as lowering the risk of deterioration associated with psychiatric comorbidities.

Push notifications were also demanded by many participants. The majority of the participants reported wanting push notifications as reminders for skill use, with a common request of sending the notifications when they were not practicing skills or engaging in ED behaviors. Reminders have been shown to be effective in improving patients’ engagement in digital interventions for EDs [33]. During the parent study, some participants received just-in-time adaptive interventions based on their self-monitoring records throughout the day, which served as reminders to use appropriate skills at high-risk times [25]. However, participants were engaging in high-intensity daily self-monitoring during treatment, which provided information on when they were most in need of skill use reminders. The same level of self-monitoring might not be feasible in posttreatment deterioration prevention, as participants in this study also mentioned that weekly self-monitoring posttreatment would be just enough, and daily might be too burdensome. A potential solution could be to use wearable passive sensors to detect the optimal time to deliver reminders for skill use. Previous research has demonstrated that passive sensors could be used to measure ED behaviors and risk factors associated with eating pathology (eg, negative affect and dietary restriction) [34]. Therefore, given the well-established utility of and patients’ preferences, push notifications should be included as a feature in digital deterioration prevention to serve as reminders for skill use. Future research is needed to examine whether passive sensors could be incorporated into deterioration prevention to detect the optimal timing and content of the push notifications without having patients self-monitor intensively.

Participants also expressed a need for human connection within the app. Most wanted to connect with other users with the same diagnosis so that they could learn from and support each other, reflecting the importance of social support in the recovery process [6,12]. Mobile interventions could overcome geographical barriers and make it easier to connect patients with similar experiences, and thus provide social support that patients themselves might have difficulty accessing [35]. Therefore, having patients who are using the app connect with each other could be a potential feature to include in digital deterioration prevention to foster a supportive social support environment. Additionally, participants also expressed an interest in connecting with professionals as needed in the app, especially when they are in an emergency or crisis. Lack of access to mental health services due to financial restraints, difficulty navigating through the health care system, or other reasons has been shown to be a contributor to posttreatment deterioration in patients with BN-EDs [12]. Previous research has demonstrated that therapist involvement in ED digital interventions might improve patients’ satisfaction [36], which is tightly associated with engagement [21]. However, including licensed therapists or other mental health providers in digital interventions might significantly increase the cost and thus impede dissemination [37-39]. Trained coaches who are not licensed professionals could be a potential alternative, given that they have been shown to be effective in digital interventions and that they could be more cost-effective [30,40,41]. Including a human connection component in digital deterioration prevention for patients with BN-EDs could potentially lower the risk of deterioration by providing social support and improving patient engagement with the app. To optimize treatment engagement and outcomes, future research should examine which specific or combination of types of human connection would be the most beneficial.

By implementing the UCD approach and actively involving patients in the design process through interviews, the study directly captured and incorporated feedback on patients’ desired features and potential functionality, ensuring that the final design of the product will reflect core users’ needs and preferences.

### Limitations

This study has several limitations. First, this study only included participants who received outpatient CBT-E in a clinical trial so they needed to follow a strict timeline of finishing treatment (ie, within 16 weeks) and received treatment based on standardized treatment manuals that clinicians could rarely deviate from which only represented certain subgroups of the broader population with BN-EDs receiving CBT-E (ie, patients receiving CBT-E outside of a clinical trial). Second, the average follow-up period from EOT to this study was 39.85 (SD 3.95) months, and participants were asked to think back to the time when they just finished treatment and were asked questions with specific ED terms, such as LOC eating and compensatory behaviors, which could lead to recall bias and difficulty comprehending the questions. Third, related to the long follow-up period, participants’ needs could change over time, and this study did not capture this change. Fourth, participants from this study were selected because they voluntarily expressed interest in participating in this follow-up study, which could indicate that they might be naturally more compliant and enthusiastic about CBT-E or ED treatment in general compared to others who did not respond to the recruitment email. Therefore, selection bias could also be a limitation. Similarly, the majority of the participants experienced symptom deterioration during the follow-up period, which might be a motivator for them to participate in this study. As a result, this study did not capture the perspectives of those who have not deteriorated yet after treatment ended.

### Conclusions

This study conducted a needs assessment to explore the interests and preferences of patients with BN-EDs for post-CBT-E digital deterioration prevention. Insights from participants highlighted the perceived importance of continued support for consistent skill use and provided valuable implications about what features should be included in digital deterioration prevention programs. Future research should examine the optimal approaches to deliver the core features identified in this study that could lead to higher continued skill use and a lower risk of deterioration in the long term.

## Appendix

Multimedia Appendix 1 Visuals of app features presented to participants during the interview.

## Abbreviations

BN-ED: bulimia-spectrum eating disorder
CBT-E: enhanced cognitive behavioral therapy
EDE: eating disorder examination
EOT: end of treatment
LOC: loss of control
UCD: user-centered design

## Data Availability

The datasets generated during or analyzed during this study are available from the corresponding author upon reasonable request.

## References

[1] Fitzsimmons-Craft EE, Ciao AC, Accurso EC, Pisetsky EM, Peterson CB, Byrne CE, Le Grange D (2014). Subjective and objective binge eating in relation to eating disorder symptomatology, depressive symptoms, and self-esteem among treatment-seeking adolescents with bulimia nervosa. Eur Eat Disord Rev.

[2] Fairburn CG, Cooper Z, Doll HA, O'Connor ME, Bohn K, Hawker DM, Wales JA, Palmer RL (2009). Transdiagnostic cognitive-behavioral therapy for patients with eating disorders: a two-site trial with 60-week follow-up. Am J Psychiatry.

[3] Linardon J, Wade TD (2018). How many individuals achieve symptom abstinence following psychological treatments for bulimia nervosa? A meta-analytic review. Int J Eat Disord.

[4] Södersten P, Bergh C, Leon M, Brodin U, Zandian M (2017). Cognitive behavior therapy for eating disorders versus normalization of eating behavior. Physiol Behav.

[5] Olmsted MP, Kaplan AS, Rockert W (2005). Defining remission and relapse in bulimia nervosa. Int J Eat Disord.

[6] Olmsted MP, MacDonald DE, McFarlane T, Trottier K, Colton P (2015). Predictors of rapid relapse in bulimia nervosa. Int J Eat Disord.

[7] Ricca V, Castellini G, Mannucci E, Lo Sauro C, Ravaldi C, Rotella CM, Faravelli C (2010). Comparison of individual and group cognitive behavioral therapy for binge eating disorder. A randomized, three-year follow-up study. Appetite.

[8] Stewart SL, Kam C, Baiden P (2014). Predicting length of stay and readmission for psychiatric inpatient youth admitted to adult mental health beds in Ontario, Canada. Child Adolesc Ment Health.

[9] Clancy OM, Juarascio AS, Manasse SM, Srivastava P (2023). Predicting cognitive-behavioral therapy outcomes for bulimia nervosa patients based on skill use during treatment. J Behav Cogn Ther.

[10] D'Adamo L, Linardon J, Manasse SM, Juarascio AS (2024). Trajectories of therapeutic skills use and their dynamic relations to symptom change during cognitive-behavioral therapy for bulimia nervosa. Int J Eat Disord.

[11] Srivastava P, Felonis C, Lin M, Clarke K, Juarascio A (2023). Reciprocal association between session-by-session change in overvaluation of shape and weight and session-by-session change in bulimia nervosa symptoms during cognitive behavior therapies. Eat Disord.

[12] Liu J, Wang H, Wetherall L, Giannone A, Juarascio A (2024). Patients' perceptions of post-treatment factors that influenced skill use after cognitive-behavioral therapy for bulimia nervosa spectrum disorders. Int J Eat Disord.

[13] Cockell SJ, Zaitsoff SL, Geller J (2004). Maintaining change following eating disorder treatment. Prof Psychol Res Pr.

[14] Woodside DB, Kohn M, Kerr A (1998). Patterns of relapse and recovery following intensive treatment for eating disorders: a qualitative description. Eat Disord.

[15] Bauer S, Okon E, Meermann R, Kordy H (2012). Technology-enhanced maintenance of treatment gains in eating disorders: efficacy of an intervention delivered via text messaging. J Consult Clin Psychol.

[16] Fichter MM, Quadflieg N, Nisslmüller K, Lindner S, Osen B, Huber T, Wünsch-Leiteritz W (2012). Does internet-based prevention reduce the risk of relapse for anorexia nervosa?. Behav Res Ther.

[17] Gulec H, Moessner M, Mezei A, Kohls E, Túry F, Bauer S (2011). Internet-based maintenance treatment for patients with eating disorders. Prof Psychol Res Pract.

[18] Robinson S, Perkins S, Bauer S, Hammond N, Treasure J, Schmidt U (2006). Aftercare intervention through text messaging in the treatment of bulimia nervosa—feasibility pilot. Int J Eat Disord.

[19] Anastasiadou D, Folkvord F, Lupiañez-Villanueva F (2018). A systematic review of mHealth interventions for the support of eating disorders. Eur Eat Disord Rev.

[20] Hamid N (2024). Internet-based cognitive behaviour therapy for the prevention, treatment and relapse prevention of eating disorders: a systematic review and meta-analysis. Psych J.

[21] Graham AK, Lattie EG, Mohr DC (2019). Experimental therapeutics for digital mental health. JAMA Psychiatry.

[22] Graham AK, Wildes JE, Reddy M, Munson SA, Taylor CB, Mohr DC (2019). User-centered design for technology-enabled services for eating disorders. Int J Eat Disord.

[23] Lyon AR, Koerner K (2016). User-centered design for psychosocial intervention development and implementation. Clin Psychol (New York).

[24] Ayala GX, Elder JP (2011). Qualitative methods to ensure acceptability of behavioral and social interventions to the target population. J Public Health Dent.

[25] Juarascio AS, Presseller EK, Srivastava P, Manasse SM, Forman EM (2023). A randomized controlled trial of CBT+: a clinician-controlled, just-in-time, adjunctive intervention for bulimia-spectrum disorders. Behav Modif.

[26] Kim JP, Sadeh-Sharvit S, Welch HA, Neri E, Tregarthen J, Lock J (2022). Eating disorders early app use mediates treatment effect on clinical improvement. Int J Eat Disord.

[27] Carter JC, Olmsted MP, Kaplan AS, McCabe RE, Mills JS, Aimé A (2003). Self-help for bulimia nervosa: a randomized controlled trial. Am J Psychiatry.

[28] Fairburn CG, Wilson GT (1993). The eating disorder examination. Binge Eating: Nature, Assessment, and Treatment. 12th Edition.

[29] Juarascio AS, Manasse SM, Goldstein SP, Forman EM, Butryn ML (2015). Review of smartphone applications for the treatment of eating disorders. Eur Eat Disord Rev.

[30] Graham AK, Kosmas JA, Massion TA (2023). Designing digital interventions for eating disorders. Curr Psychiatry Rep.

[31] O’Connor C, Joffe H (2020). Intercoder reliability in qualitative research: debates and practical guidelines. Int J Qual Methods.

[32] Keel PK, Dorer DJ, Franko DL, Jackson SC, Herzog DB (2005). Postremission predictors of relapse in women with eating disorders. Am J Psychiatry.

[33] Anastasiadou D, Folkvord F, Serrano-Troncoso E, Lupiañez-Villanueva F (2019). Mobile health adoption in mental health: user experience of a mobile health app for patients with an eating disorder. JMIR Mhealth Uhealth.

[34] Presseller EK, Patarinski AGG, Fan SC, Lampe EW, Juarascio AS (2022). Sensor technology in eating disorders research: a systematic review. Int J Eat Disord.

[35] Tregarthen JP, Lock J, Darcy AM (2015). Development of a smartphone application for eating disorder self-monitoring. Int J Eat Disord.

[36] Aardoom JJ, Dingemans AE, Spinhoven P, van Ginkel JR, de Rooij M, van Furth EF (2016). Web-based fully automated self-help with different levels of therapist support for individuals with eating disorder symptoms: a randomized controlled trial. J Med Internet Res.

[37] Bernstein EE, Weingarden H, Wolfe EC, Hall MD, Snorrason I, Wilhelm S (2022). Human support in app-based cognitive behavioral therapies for emotional disorders: scoping review. J Med Internet Res.

[38] McClure Z, Fuller-Tyszkiewicz M, Messer M, Linardon J (2024). Predictors, mediators, and moderators of response to digital interventions for eating disorders: a systematic review. Int J Eat Disord.

[39] Provoost S, Kleiboer A, Ornelas J, Bosse T, Ruwaard J, Rocha A, Cuijpers P, Riper H (2020). Improving adherence to an online intervention for low mood with a virtual coach: study protocol of a pilot randomized controlled trial. Trials.

[40] Nitsch M, Dimopoulos CN, Flaschberger E, Saffran K, Kruger JF, Garlock L, Wilfley DE, Taylor CB, Jones M (2016). A guided online and mobile self-help program for individuals with eating disorders: an iterative engagement and usability study. J Med Internet Res.

[41] Fitzsimmons-Craft EE, Taylor CB, Graham AK, Sadeh-Sharvit S, Balantekin KN, Eichen DM, Monterubio GE, Goel NJ, Flatt RE, Karam AM, Firebaugh M, Jacobi C, Jo B, Trockel MT, Wilfley DE (2020). Effectiveness of a digital cognitive behavior therapy-guided self-help intervention for eating disorders in college women: a cluster randomized clinical trial. JAMA Netw Open.

