# Supplementary material for: Understanding Patients’ Preferences for a Digital Intervention to Prevent Posttreatment Deterioration for Bulimia-Spectrum Eating Disorders: User-Centered Design Study
Source: JMIR Form Res. 2024 Nov 18;8:e60865. doi: 10.2196/60865 (PMC11612586; doi:10.2196/60865)
Supplement: Multimedia Appendix 1 [file formative_v8i1e60865_app1.pptx]

## Slide 1
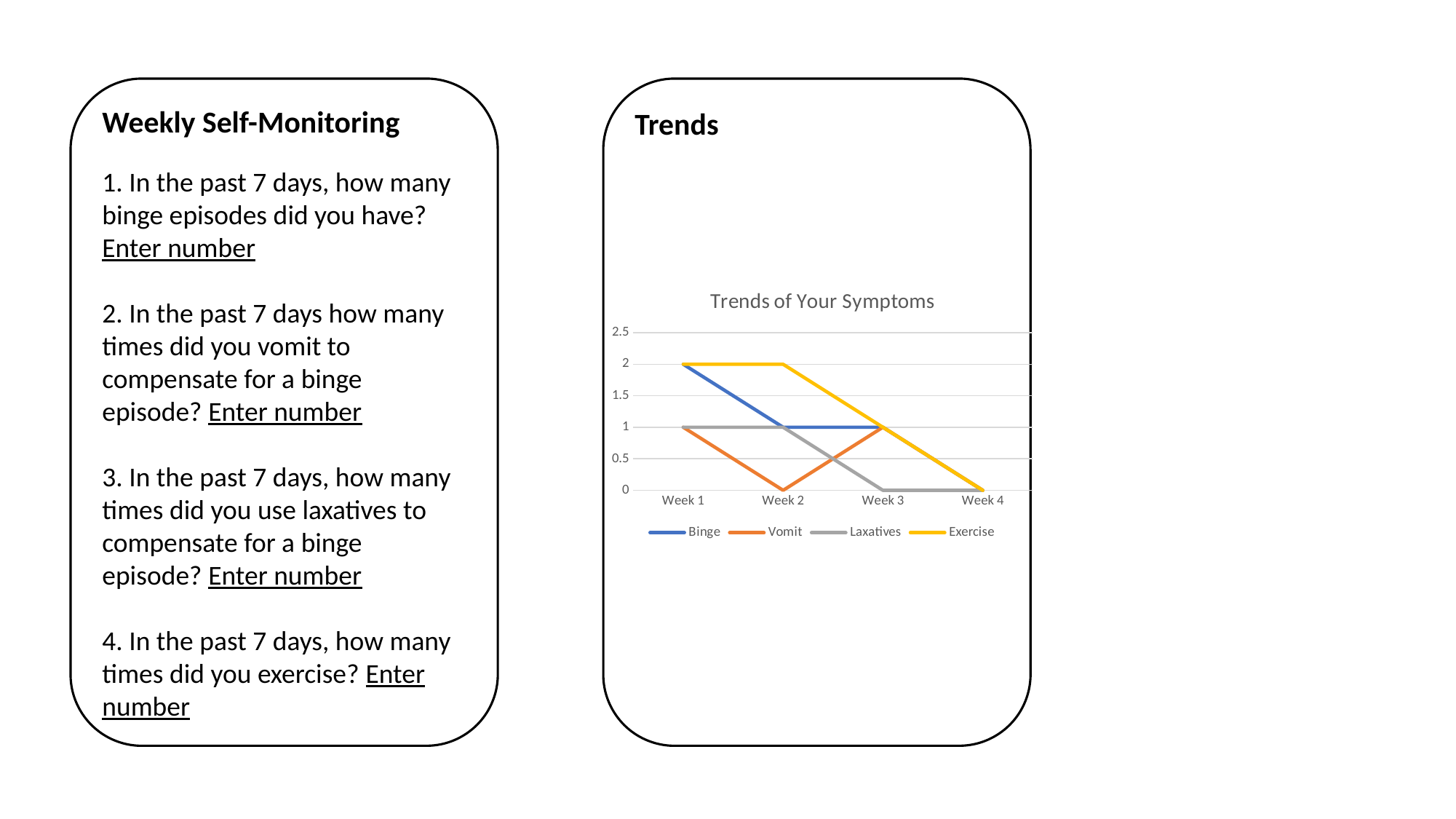

Weekly Self-Monitoring
1. In the past 7 days, how many binge episodes did you have? Enter number
2. In the past 7 days how many times did you vomit to compensate for a binge episode? Enter number
3. In the past 7 days, how many times did you use laxatives to compensate for a binge episode? Enter number
4. In the past 7 days, how many times did you exercise? Enter number
Trends
### Chart: Trends of Your Symptoms
| Category | Binge | Vomit | Laxatives | Exercise |
|---|---|---|---|---|
| Week 1 | 2.0 | 1.0 | 1.0 | 2.0 |
| Week 2 | 1.0 | 0.0 | 1.0 | 2.0 |
| Week 3 | 1.0 | 1.0 | 0.0 | 1.0 |
| Week 4 | 0.0 | 0.0 | 0.0 | 0.0 |

## Slide 2
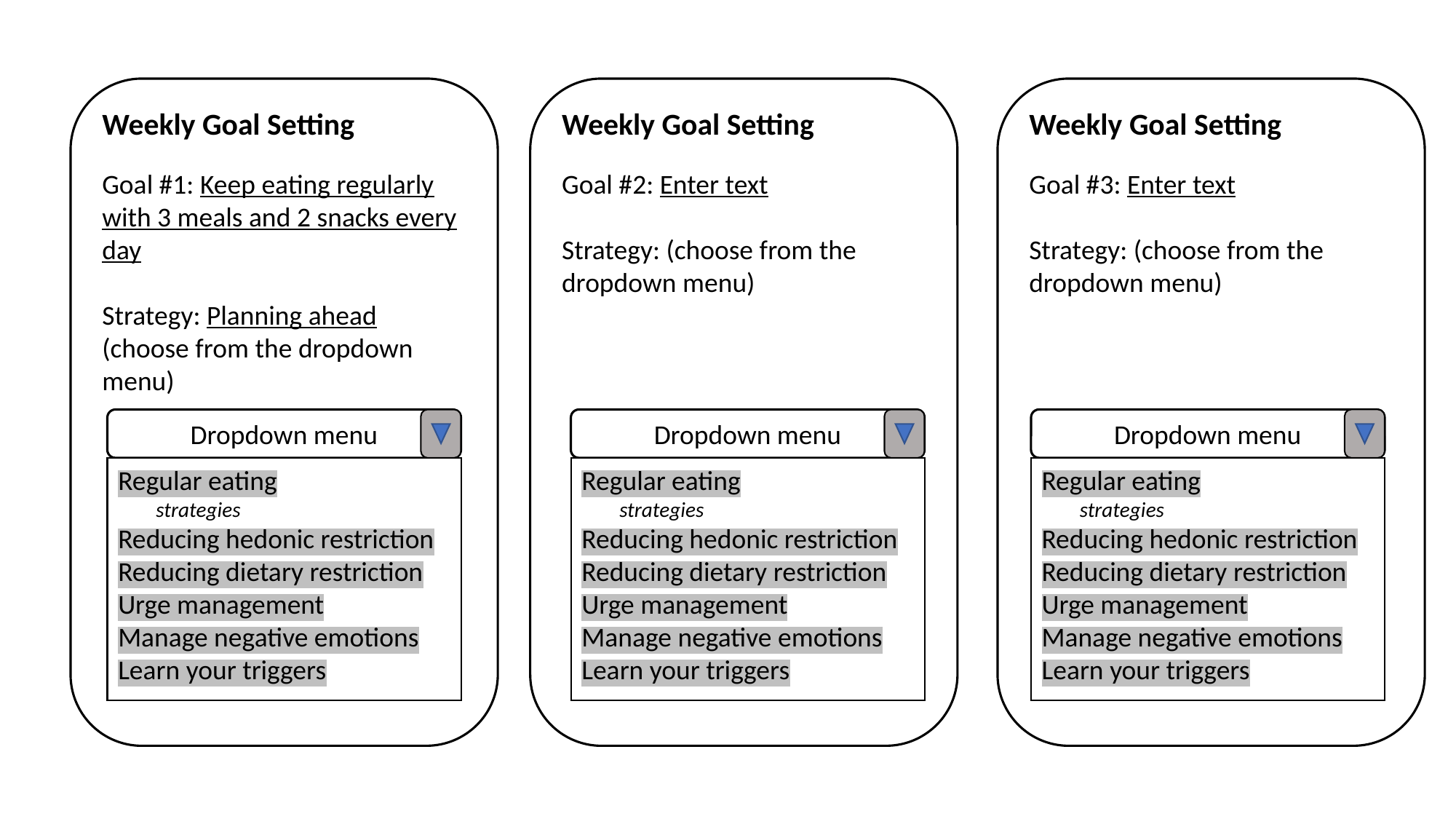

Weekly Goal Setting
Goal #3: Enter text
Strategy: (choose from the dropdown menu)
Weekly Goal Setting
Goal #1: Keep eating regularly with 3 meals and 2 snacks every day
Strategy: Planning ahead (choose from the dropdown menu)
Weekly Goal Setting
Goal #2: Enter text
Strategy: (choose from the dropdown menu)
Dropdown menu
Dropdown menu
Dropdown menu
Regular eating
 strategies
Reducing hedonic restriction
Reducing dietary restriction
Urge management
Manage negative emotions
Learn your triggers
Regular eating
 strategies
Reducing hedonic restriction
Reducing dietary restriction
Urge management
Manage negative emotions
Learn your triggers
Regular eating
 strategies
Reducing hedonic restriction
Reducing dietary restriction
Urge management
Manage negative emotions
Learn your triggers

## Slide 3
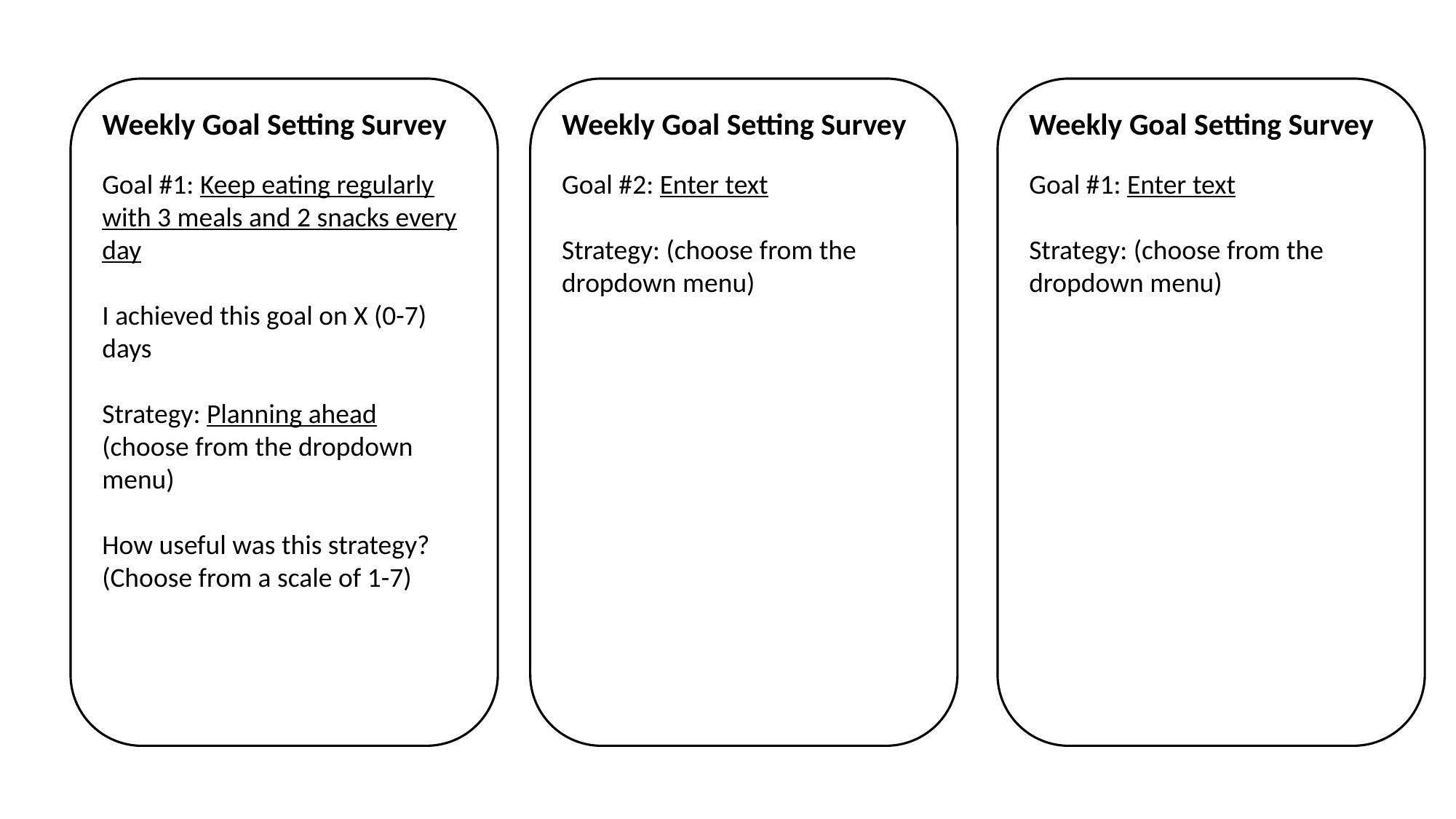

Weekly Goal Setting Survey
Goal #1: Enter text
Strategy: (choose from the dropdown menu)
Weekly Goal Setting Survey
Goal #1: Keep eating regularly with 3 meals and 2 snacks every day
I achieved this goal on X (0-7) days
Strategy: Planning ahead (choose from the dropdown menu)
How useful was this strategy? (Choose from a scale of 1-7)
Weekly Goal Setting Survey
Goal #2: Enter text
Strategy: (choose from the dropdown menu)

## Slide 4
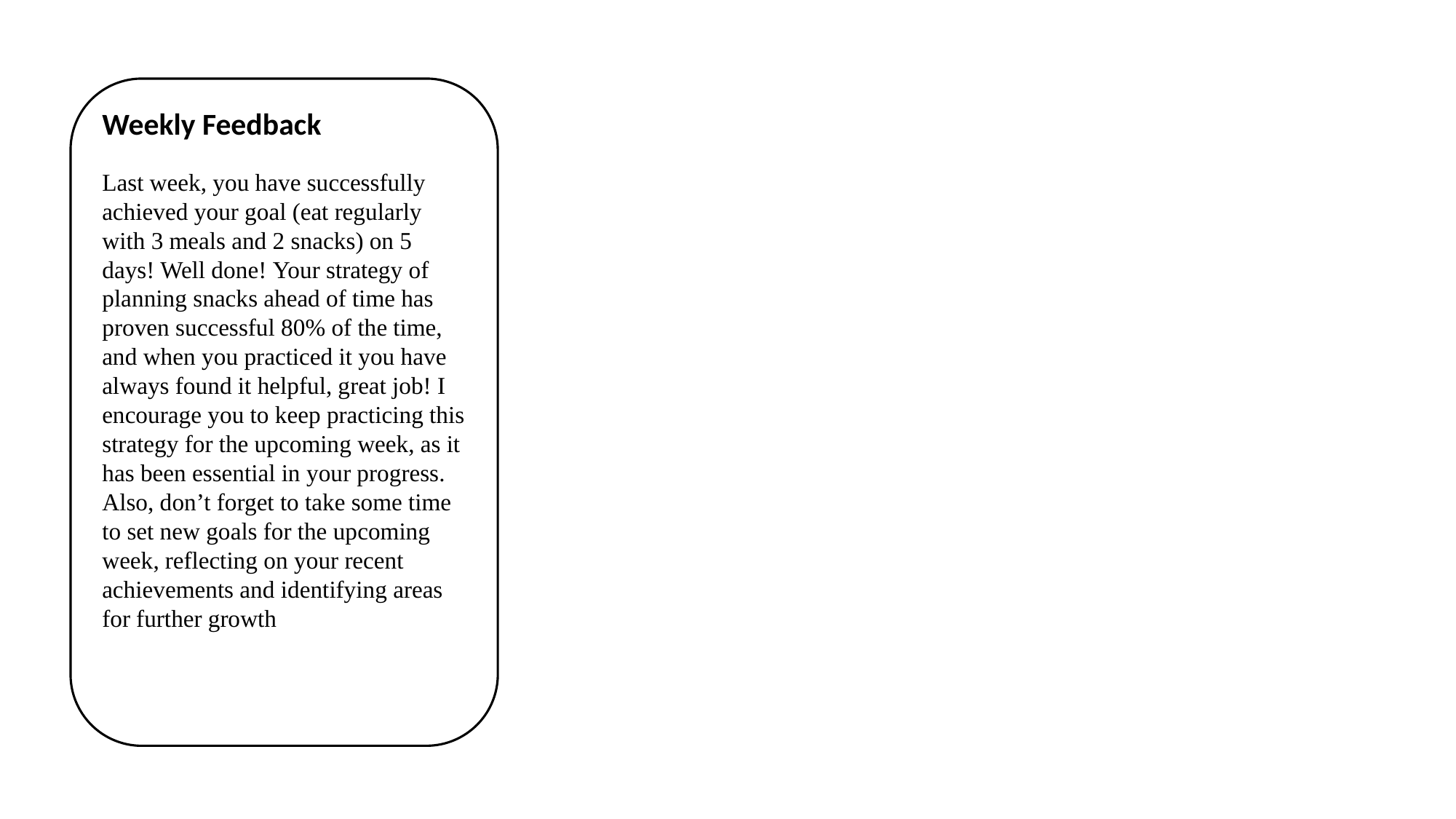

Weekly Feedback
Last week, you have successfully achieved your goal (eat regularly with 3 meals and 2 snacks) on 5 days! Well done! Your strategy of planning snacks ahead of time has proven successful 80% of the time, and when you practiced it you have always found it helpful, great job! I encourage you to keep practicing this strategy for the upcoming week, as it has been essential in your progress. Also, don’t forget to take some time to set new goals for the upcoming week, reflecting on your recent achievements and identifying areas for further growth

## Slide 5
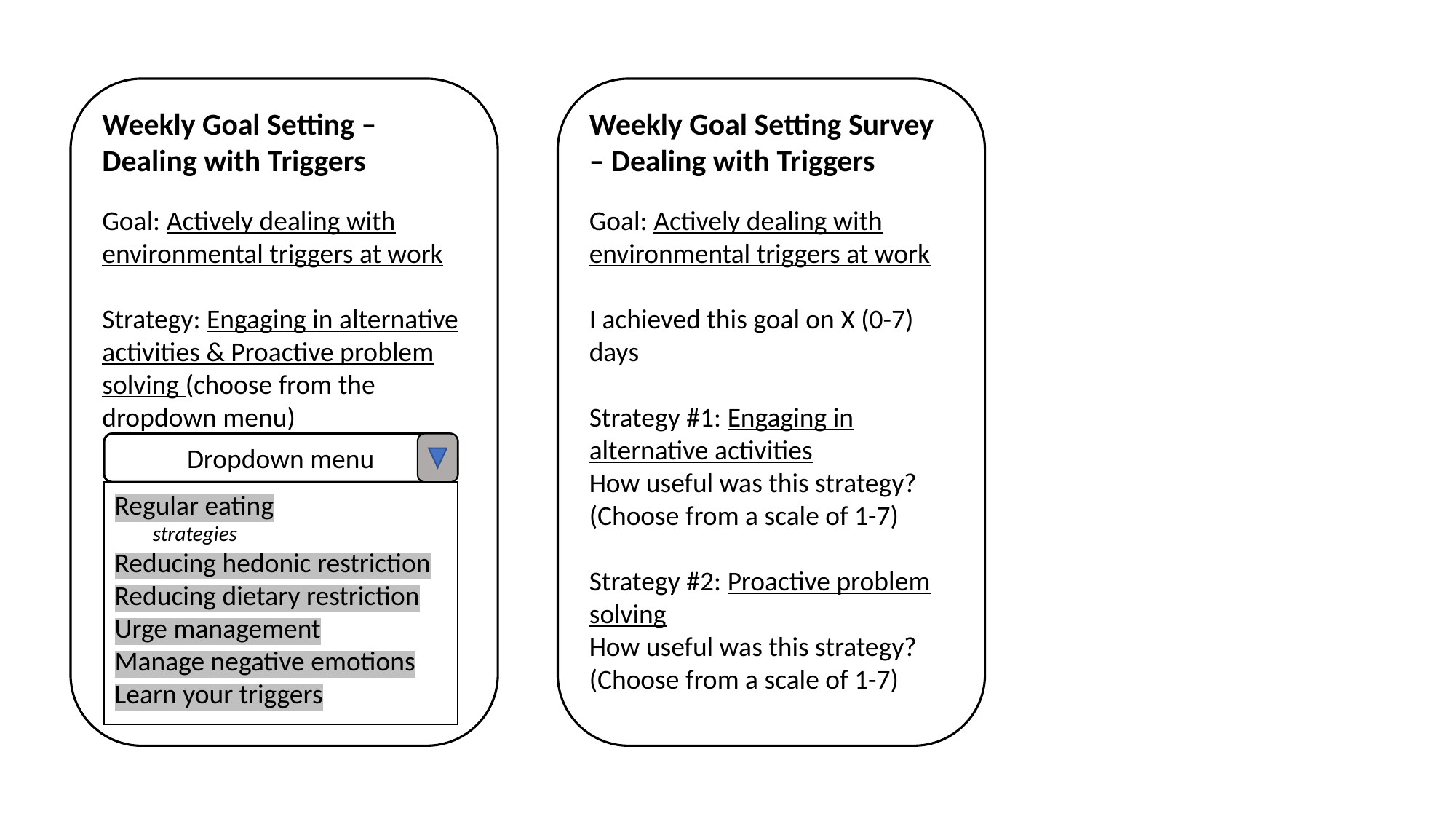

Weekly Goal Setting – Dealing with Triggers
Goal: Actively dealing with environmental triggers at work
Strategy: Engaging in alternative activities & Proactive problem solving (choose from the dropdown menu)
Weekly Goal Setting Survey – Dealing with Triggers
Goal: Actively dealing with environmental triggers at work
I achieved this goal on X (0-7) days
Strategy #1: Engaging in alternative activities
How useful was this strategy? (Choose from a scale of 1-7)
Strategy #2: Proactive problem solving
How useful was this strategy? (Choose from a scale of 1-7)
Dropdown menu
Regular eating
 strategies
Reducing hedonic restriction
Reducing dietary restriction
Urge management
Manage negative emotions
Learn your triggers
